# Diphtheria outbreak in Yemen: the impact of conflict on a fragile health system

**DOI:** 10.1186/s13031-019-0204-2

**Published:** 2019-05-22

**Authors:** Fekri Dureab, Maysoon Al-Sakkaf, Osan Ismail, Naasegnibe Kuunibe, Johannes Krisam, Olaf Müller, Albrecht Jahn

**Affiliations:** 1 Heidelberg Institute of Global Health, Hospital University- Heidelberg, Heidelberg, Germany; 2 World Health Organization, WHO, Yemen Country Office, Sana’a, Yemen; 3 University for Development Studies, Tamale, Ghana; 4 Institute of Medical Biometry and Informatics, Heidelberg University, Heidelberg, Germany

**Keywords:** Diphtheria outbreak, Immunization, Conflict, Yemen

## Abstract

**Background:**

War in Yemen started three years ago, and continues unabated with a steadily rising number of direct and indirect victims thus leaving the majority of Yemen’s population in dire need of humanitarian assistance. The conflict adversely affects basic socioeconomic and health conditions across the country.

**Methods:**

This study analyzed the recent ongoing diphtheria outbreak in Yemen and in particular, the health system’s failure to ensure immunization coverage and respond to this outbreak. Data from the weekly bulletins of the national electronic Disease Early Warning System’s (eDEWS) daily diphtheria reports and district immunization coverage were analyzed. The number of diphtheria cases and deaths, and immunization coverage (DPT) were reviewed by district including the degree to which a district was affected by conflict using a simple scoring system. A logistic regression and bivariate correlation were applied using the annual immunization coverage per district to determine if there was an association between diphtheria, immunization coverage and conflict.

**Results:**

The study results confirm the association between the increasing cases of diphtheria, immunization coverage and ongoing conflict. A total of 1294 probable cases of diphtheria were reported from 177 districts with an overall case fatality rate of 5.6%. Approximately 65% of the patients were children under 15 years, and 46% of the cases had never been vaccinated against diphtheria. The risk of an outbreak increased by 11-fold if the district was experiencing ongoing conflict *p* < 0.05. In the presence of conflict (whether past or ongoing), the risk of an outbreak decreased by 0.98 if immunization coverage was high *p* > 0.05.

**Conclusion:**

The conflict is continuously devastating the health system in Yemen with serious consequences on morbidity and mortality. Therefore, the humanitarian response should focus on strengthening health services including routine immunization procedures to avoid further outbreaks of life-threatening infectious diseases, such as diphtheria.

## Introduction

Diphtheria is a life-threatening bacterial disease caused by *Corynebacterium diphtheria*, a non-encapsulated gram-positive bacillus. It is transmitted through close respiratory contact, causes airway obstruction due to nasopharyngeal infection, and may spread to other organs [1–3].

Diphtheria is a vaccine-preventable disease, which was largely eliminated in industrialized countries decades ago. In low-income countries, diphtheria control was much improved by global efforts, such as the Expanded Program on Immunization (EPI) in the second half of the twentieth century [4]. However, diphtheria re-emerged during the 1990s in a number of countries In Europe, triggered by the breakdown of health services across the former Soviet Union [2, 5].

Diphtheria remains a problem in a number of low-income countries with poor immunization coverage. Several outbreaks have been reported in sub-Saharan Africa (e.g. Nigeria and Madagascar) since 2000 [6]. Bangladesh experienced recently an outbreak in a large refugee camp for the Rohinga in 2017 [7]. Currently, India, Indonesia and Nepal have the highest number of diphtheria cases in Asia [6].

Even in countries with rather good immunization coverage, such as Thailand and Iran, outbreaks of 157 and 513 cases respectively, have occurred in recent years [6]. Since the last major outbreaks of diphtheria in the 1990s, cases continue to be reported from Europe as well. In 2014, for example, 22 cases of confirmed diphtheria were reported in the European Union, and about half of these cases were in Latvia [8].

Yemen had experienced no serious diphtheria outbreaks until very recently. From October 2017 to August 2018, 2203 probable diphtheria cases (including 116 deaths) were reported. Unfortunately, few diphtheria case alerts were generated prior to the declaration of an outbreak by the electronic surveillance system [9]. Yemen has been engaged in civil war since March 2015, which has severely affected the country’s infrastructure including health services. Less than 50% of existing health facilities are fully functional and there is a serious shortage of staff, medicine and equipment [10]. The conflict has led to major population movements, increased direct morbidity and mortality, and indirect adverse effects on the population due to dysfunctional services and a lack of food, clean water, and sanitation [11]. One consequence has been a significant cholera epidemic since 2016 [12–14].

Yemen is in the southwest of the Arabian Peninsula, bordered by Saudi Arabia to the north and Oman to the east and surrounded by water to the south and west [15]. The country is administratively divided into 22 governorates and 333 districts. It is a low-income country with high poverty and illiteracy rates [16]. The country has experienced many crises since 2011, which began with the Arab Spring’s efforts against poverty, unemployment, corruption, and political instability. The political situation moved into a new complicated stage in March 2015 with the beginning of civil war [17], which has led to the country’s fragmentation into multiple semi-autonomous entities running basic services [18, 19].

The health systems include four levels of health facilities: health units, health centers, district or governorate hospitals, and referral hospitals [20]. There are approximately 4207 public health facilities including 243 hospitals [21]. Approximately 16.4 million have no access to basic healthcare [22], and only 43% of the functional health facilities have communicable diseases services. Maternal and new-born services including immunization services are available in only 35% of functional health facilities [23]. Diphtheria outbreaks reflect a huge gap in the immunization coverage in the last three years due to the obviously collapsed health system in Yemen. A recent WHO report shows that the coverage for vaccination against diphtheria/pertussis/tetanus 1 (DPT1) coverage shrank gradually over the last three years: approximately 89, 88 and 83% in 2015, 2016 and 2017 respectively [24, 25]. This paper describes the recent diphtheria outbreak and explains the relationship between diphtheria cases, immunization and conflict dynamics in Yemen.

## Methods

### Data sources

We used multiple national-level data sources to describe and analyze the recent diphtheria outbreak in Yemen. First, data from the weekly bulletins of the electronic Disease Early Warning System (eDEWS) (1st epidemiological week of 2017 to the 10th epidemiological week of 2018) were used to identify the trend of the diphtheria outbreak. The second data source was the daily diphtheria surveillance reports on district and governorate levels. The third source was the 2017 annual immunization coverage report from 333 Yemeni districts. Finally, the 2017 report on the level of conflict was analyzed after districts were categorized according to conflict dynamic: 1 = experience ongoing armed conflict (65 districts), 2 = history of past armed conflict (48 districts), and 3 = no conflict (219 districts). The primary outcome, “diphtheria outbreak” (yes/no) was determined for each district by assessing the presence of diphtheria cases within a district. If there was at least one case of diphtheria in a particular district, this was considered as an outbreak.

#### Diphtheria case definitions [26]

##### Clinical description

*An illness characterized by laryngitis or pharyngitis or tonsillitis, and an adherent membrane of the tonsils, pharynx and/or nose.*

##### Laboratory criteria for diagnosis

*Isolation of Corynebacterium diphtheriae from a clinical specimen, or fourfold or greater rise in serum antibody (but, only if both serum samples were obtained before the administration of diphtheria toxoid or antitoxin).*

##### Case classification

*Suspected: Not applicable.*

*Probable: A case that meets the clinical description.*

*Confirmed: A probable case that is laboratory confirmed or linked epidemiologically to a laboratory confirmed case.*

### Data analyses

We describe the trend of diphtheria cases between the 1st week of 2017 to the 10th week of 2018 in Yemen based on data obtained from the weekly epidemiological eDEWS Bulletin. To determine the relationship between the outbreak, immunization and conflict dynamics, we regressed conflict dynamic on immunization on diphtheria for all districts in 2017, whether or not the district had experienced conflict in the past year, was currently experiencing armed conflict, or had never experienced armed conflict. We tested for an interaction between immunization and past armed conflict (*imcoconf1*) and between immunization and current armed conflict (*imcoconf2*).

Given that the outcome variable (diphtheria outbreak denoted as *dipht*) was binary, we used the binary logit regression [27]. Following applications in health research [28], we specified the model as

1 $$ L=\ln \left(\frac{P_i}{1-{P}_i}\right)=\forall + X\beta +\epsilon $$

where $\mathrm{in}\left(\frac{P_i}{1-{P}_i}\right)$ is the natural logarithm of the ratio of the probability that a diphtheria outbreak will occur in a location given the explanatory variables of the respective district (P_i = P[dipht = 1|X_i]) divided by the probability that an outbreak will not occur (1-P_i), and ∀ is a constant term, *X* is a vector of explanatory variables, *β* is a vector of coefficients and *ϵ* is the random error term. Table 1 presents the definitions of all variables.

**Table 1:** Measurement of variables for analysis of diphtheria, conflict and immunization status

Variable	Definition	Measurement
*dipth*	Diphtheria outbreak in district	1 = *yes*; 0 = *no*
*imco*	Immunized children in district (# immunized children-less than 1 year old in 2017)	Continuous
*confhis*1	District experienced conflict in the past (more than 3 years)	1 = *yes*; 0 = *no*
*confhis*2	District currently experiencing conflict	1 = *yes*; 0 = *no*
*imcoconf*1	Immunized children in district with past conflict (*imco x conf his1*)	> 0
*imcoconf*2	Immunized children in district with current conflict (*imco x conf his2*)	> 0

We estimated the eq. (2) and calculated the odd ratios (OR) to compare the relative odds of a diphtheria outbreak with the given conflict dynamics while controlling for number of immunized children in the district.

2 $$ \ln \left(\frac{P_i}{1-{P}_i}\right)=\forall +{\beta}_1 imco+{\beta}_2 confhis0+{\beta}_3 confhis1+{\beta}_4 confhis2+{\beta}_5 imcoconf1+{\beta}_6 imcoconf2+\epsilon $$

OR = 1 means the particular conflict dynamic does not affect the odds of a diphtheria outbreak, OR > 1 means a particular conflict dynamic is associated with higher odds of a diphtheria outbreak, and OR < 1 means a particular conflict dynamic is associated with lower odds of a diphtheria outbreak. The variable *confhis*0 serves as base category for *confhis*1 and *confhis*2 and so does not enter the model. A *p*-value smaller than 0.05 was regarded as statistically significant. Analyses were conducted using the software Stata version 15.

## Results

A diphtheria outbreak was announced on 29 October 2017 by the Ministry of public Health and population and WHO in Yemen. From that date to March 10, 2018, a total of 1294 probable cases were recorded in 177/333 (53%) districts in 20/23 (87%) governorates. Table 2 presents the distribution of reported cases, deaths and corresponding case fatality rate (CFR) by governorates. Most cases occurred in three governorates, Ibb governorate (441cases, 34%), Hodeida governorate (151 cases, 12%) and Sana’a governorate (133 cases, 10%). A total of 73 deaths were reported in all governorates, which resulted in an overall CFR of 5.6%.

**Table 2:** Distribution of diphtheria cases, deaths and corresponding case fatality rates by governorates in Yemen (October 2017 – March 2018)

Governorates	Total No. of districts	No of affected districts	No of probable cases	Deaths	CFR (%)
Ibb	20	19	441	16	3.6
Abyan	11	1	4	2	50.0
Sana’a city	10	9	60	1	1.7
Al Baidha	20	11	19	3	15.8
Al Jawf	12	3	6	3	50.0
Al Hodeida	26	20	151	12	7.9
Ad Dhale‘a	9	8	107	2	1.9
Al Mahweet	9	8	52	1	1.9
Al Mahrah	9	0	0	0	
Taiz	23	15	45	6	13.3
Hajjah	31	15	48	4	8.3
Al Mukalla	12	0	0	0	
Say’on	16	2	2	0	0.0
Damar	12	9	39	4	10.3
Raymah	6	3	6	2	33.3
Socotra	2	0	0	0	
Shabwah	17	1	1	0	0.0
Sadah	15	4	11	3	27.3
Sana’a	16	15	133	5	3.8
Aden	8	7	63	2	3.2
Amran	20	19	86	4	4.7
Lahj	15	5	10	2	20.0
Mareb	14	3	10	1	10.0
Total	333	177	1294	73	5.6%

**Fig. 1:**
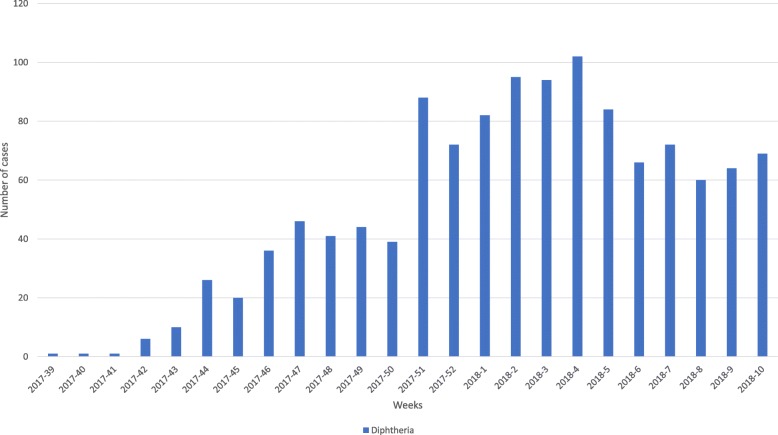
Trend of the diphtheria probable cases from epidemiological week 39 in 2017 to week 10 in 2018 in Yemen

Figure 1 shows the trend in reported number of probable diphtheria cases and alerts generated by eDEWS, from epidemiological week 39 in 2017 to week 10 in 2018. The trend shows a gradual increase in diphtheria cases reaching a peak in epidemiological week 4 in 2018 (102 cases/week; reported by 14 governorates). The trend then shows a gradual decline in the number of cases.

Table 3 shows the distribution of diphtheria cases and deaths and corresponding CFRs by age, sex and vaccination status. Diphtheria morbidity and mortality did not significantly differ between males and females and the majority of cases occurred in children and adolescents who also had the highest CFRs (11% in children under 5 years old, but only approximately 1% in those 35–50 years old). Diphtheria vaccination status was strongly and inversely associated with CFRs. However, although 31% of diphtheria cases were people reported to have taken three doses of diphtheria vaccination, their CFR was only 2.9%.

**Table 3:** Distribution of diphtheria cases and deaths and corresponding CFRs by age, sex and vaccination status

Variable	Reported cases		Deaths		CFR
	Frequency	Percent	Frequency	Percent	Percent
*Sex*					
Male	673	52	37	51	5.5
Female	621	48	36	49	5.8
*Age groups*					
0–4 years	259	20	29	40	11
5–9 years	324	25	21	29	6
10–14 years	259	20	13	18	5
15–24 years	194	15	7	10	7.7
25–34 years	129	10	1	1	0.8
35–50 years	91	7	1	1	1
> 50 years	39	3	1	1	2.5
*Vaccination status*					
0 dose	595	46	50	69	8.4
1 dose	26	2	2	3	7.7
2 doses	78	6	4	5	5.1
3 doses	401	31	12	16	2.9
Unknown	194	15	5	7	2.6

### Relationship between conflict and diphtheria outbreak

Table 4 shows the results from the bivariate logistic regression analysis. To explore the relationship between the conflict situation in Yemen and the diphtheria outbreak, we regressed the outcome variable (whether a district experienced outbreak or not as diphtheria outbreak = 1, no outbreak = 0) with the conflict dynamic in the district. The bivariate regression results show that immunization generally does affect the probability of diphtheria outbreak (OR = 1.02; CI = 1.01–1.03, *p* < 0.05). The same was observed for the effects of immunization in areas where conflict was ongoing (OR = 1.01; CI = 1.004–1.016, *p* < 0.05). However, the probability of an outbreak increased significantly in areas where conflict was ongoing (OR = 1.89; CI = 1.20–2.99, *p* < 0.05). From the multivariate regression results, immunization did affect the risk of an outbreak (OR = 1.04; CI =1.012–1.058, *p* < 0.05). This is to say, that in a district without conflict, immunization had a minimal effect on the diphtheria outbreak situation. The odds of an outbreak significantly increased 11-fold if the district was experiencing ongoing conflict (OR = 11.21; CI =1.29–97.69, *p* < 0.05) and 3-fold if the district had had a history of conflict in the past year (although not statistically significant) see Table 5.

**Table 4:** Bivariate logistic regression results for number of immunized children and conflict

Variable	Odds ratio (OR)	*P*-value	95% CI
Number of immunized children	1.02***	0.001	1.01–1.03
Conflict in past year	0.59*	0.06	0.33–1.04
Ongoing conflict	1.89***	0.01	1.20–2.99
Number of immunized children in areas with past conflict	0.99**	0.16	0.99–1.00
Number of immunized children in areas with ongoing conflict	1.01**	0.001	1.00–1.02

*N* = 333 Significance: * 10% level; ** 5% level; *** 1% level.

**Table 5:** Multivariate logistic regression results for immunization and conflict

Variable	OR	*P*-value	95% CI
Number of immunized children	1.04	0.002	1.01–1.058
Conflict in past year	3.25	0.45	0.16–67.70
Ongoing conflict	11.21	0.03	1.29–97.69
Number of immunized children in areas with past conflict	0.98	0.32	0.95–1.02
Number of immunized children in areas with ongoing conflict	0.98	0.13	0.95–1.01
Constant	0.05	0.003	0.01–0.35

## Discussion

The number of cases detected weekly by eDEWS revealed that a number of diphtheria alerts had already been detected in 2017, starting in epidemiological week 5. An official statement of the Ministry of Public Health and Population (MOPHP) on the outbreak was only launched in epidemiological week 39. This gap can be explained by the intensity of the current war that delays the timeliness of reporting and response and increases the inaccessibility to basic services [14]. Children under 15 years were the most affected during this diphtheria outbreak (65%) compared to those in older age groups. For comparison, a similar study in Lao People’s Democratic Republic (Laos), in 2016, revealed that 69% of diphtheria cases were among children under 15 years [29]. The overall diphtheria CFR in Yemen has been 5.6%, and was highest among under-five children (11%). Likewise, during the recent diphtheria outbreak among the Rohingya refugee in Bangladesh, 13% of affected cases were children under five [30].

In our study, 46% of diphtheria cases and 69% of deaths were among the unvaccinated group. This corresponds to findings from a case-control study in Laos where approximately 34% of the people with diphtheria had not received any DPT doses [29, 31].

This study revealed the relationship between conflict and the diphtheria outbreak in Yemen, showing that the risk of a diphtheria outbreak significantly increases by 11-fold if the district is currently experiencing armed conflict. This supports findings from another study on this topic in Yemen [14].

There is no doubt that conflict affects the delivery of health services, since about half of the health facilities are currently not functioning in Yemen [32]. As one consequence, immunization coverage is severely affected by the ongoing conflict, which largely explains the current infectious disease outbreaks in the country. Despite much evidence on the role of immunization coverage to prevent infection [33], this study shows that in a district that does not experience conflict, immunization coverage has a minimal effect on diphtheria outbreaks. One potential explanation of this unexpected finding is the inaccuracy of coverage rates per district, since the national EPI uses the number of a total population from old census data of 2004 as denominators without considering the increasing population due to movements by internally displaced persons (IDPs) during the war. Moreover, the quality of information has likely been affected by the breakdown of national reporting mechanisms. Also, this unexpected finding might have occurred due to unmeasured, unknown confounders that were not included into the logistic regression model. Finally, the quality of the vaccines in use could be affected due to breakdowns in the cold chain.

Continuous population movement is one of the conflict-related factors that contributes to the rapid spread of infectious diseases. Unfortunately, in a domestic armed conflict, individuals are forced to leave their homes and become IDPs within the country or take refuge outside the country [34]. IDPs and host communities become more susceptible to many infectious diseases due to multiple factors such as unplanned overcrowding, hygiene problems, lack of health services, and the introduction of new infectious agents. IDPs in Yemen may thus be an important contributing factor for the diphtheria outbreak. For example, this may explain the serious situation in the Ibb governorate, which has a very large number of the IDPs in Yemen [35].

In addition to the impact of the current war that has destroyed the country’s infrastructure and deprived many people of the most basic services, the problem of IDPs and low immunization coverage, there are many additional factors that may have contributed to the emergence of epidemic diseases in Yemen over the past years. These include already existing poverty and malnutrition [36, 37]. Undernutrition has already been a huge public health problem in Yemen and contributes to the high mortality associated with infectious diseases, especially among children [38]. Approximately 2.2 million children in Yemen are acutely malnourished and 462,000 out of them suffer from severe acute malnutrition [39].

This study has some limitations. There were and still are challenges to obtain reliable data from Yemen, for example with regard to the distribution of IDPs by district. Another major limitation is related to our secondary analysis using existing poor quality data, e.g., old population counts for calculating the EPI coverage. Finally, the Yemeni health authorities have depended on patients or relatives to recall information regarding the vaccination status of cases in the daily diphtheria reports, which has a high probability of recall bias.

## Conclusion

We conclude that the conflict is continuously devastating the health system in Yemen with serious consequences on morbidity and mortality. While emergency immunization campaigns are crucial interventions in the current situation to control and prevent infectious disease outbreaks, in the long-term, Yemen needs peace and the re-establishment of functioning health services within a frame of universal health coverage.

## Abbreviations

DPT1: Diphtheria/pertussis/tetanus 1
eDEWS: Electronic Disease Early Warning System
EPI: Expanded Program on Immunization
IDPs: Internally displaced persons
MOPHP: Ministry of Public Health and Population
OR: Odd Ratio
WHO: World Health Organization
